# Deciphering the role of cathepsins in digestive system cancers: a Mendelian randomization study

**DOI:** 10.1007/s12672-025-03599-5

**Published:** 2025-09-30

**Authors:** Beian Guo, Zhiyi Zhou, Wanzhe Liao, Yurong Zheng, Yinjie Liao, Ruiqi Zeng

**Affiliations:** 1 Guangzhou Medical University, Guangzhou, 511436 China; 2 Department of Clinical Medicine, The Nanshan College of Guangzhou Medical University, Guangzhou, 511436 China; 3 Department of Clinical Medicine, The Third Clinical School of Guangzhou Medical University, Guangzhou, 511436 China; 4 Department of Clinical Medicine, The First Clinical School of Guangzhou Medical University, Guangzhou, 511436 China; 5 Shool of Computer Science and Artificial Intelligence, Wuhan University of Technology, Guangzhou, 511436 China

**Keywords:** Cathepsin, Digestive system cancers, Mendelian randomization, Biomarkers, Genetic association

## Abstract

Digestive system cancers (DSCs) present a global health challenge. This study aimed to investigate the causal relationships between cathepsins and DSCs using Mendelian Randomization (MR) analysis. Single Nucleotide Polymorphisms (SNPs) associated with nine cathepsin types (cathepsins B, E, F, G, H, O, S, L2, Z) were obtained from the INTERVAL study (3301 individuals). Genetic association data for DSCs were obtained from a large-scale GWAS conducted by Sakaue et al. (Nat Genet 53: 1415–1424, 2021), while SNPs associated with cathepsin expression were sourced from the INTERVAL study. (esophageal cancer: 998 cases, 475,308 controls; gastric cancer: 1,029 cases, 475,087 controls; colorectal cancer: 6,581 cases, 463,421 controls; hepatic cancer: 379 cases, 475,259 controls; pancreatic cancer: 1,196 cases, 475,049 controls). Inverse variance weighted (IVW), MR-Egger, and weighted median methods were adopted to conduct univariable MR (UVMR), reverse MR, and multivariable MR (MVMR) analyses to estimate causality. The UVMR analyses discovered that elevated SNP-predicted cathepsin S expression were significantly associated with increased colorectal cancer risk(IVW: *p* = 0.0162, odds ratio (OR) = 1.0513, 95% confidence interval (CI) = 1.0093–1.0951), whereas cathepsin H exhibited a protective role against pancreatic cancer(IVW: *p* = 0.0410, OR = 0.9131, 95% CI = 0.8369–0.9963). These findings were corroborated by the MVMR analyses (cathepsin S: IVW: *p* = 0.0040, OR = 1.0725, 95%CI = 1.0233–1.1252; cathepsin H: IVW: *p* = 0.0160, OR = 0.8851, 95%CI = 0.8025–0.9773). No reverse causality was found. Our findings suggested the potential causalities of cathepsins S on colorectal cancer and cathepsin H on pancreatic cancer, indicating their potential as biomarkers and therapeutic targets in DSCs. Further research is necessary to integrate these genetic associations into clinical applications.

**Supplementary Information:**

The online version contains supplementary material available at 10.1007/s12672-025-03599-5.

## Introduction

Digestive system cancers (DSCs) primarily comprise esophageal cancer, gastric cancer, colorectal cancer, hepatic cancer, and pancreatic cancer. Accounting for 25.8% of the global cancer incidence and 35.4% of cancer-related deaths, DSCs are considered to be the most prevalent malignancies and a major public health concern globally [1, 2]. While risk factors for DSCs such as obesity and alcohol consumption are well-documented, emerging research is beginning to explore novel factors [3, 4]. Among these, cathepsins are gaining attention for their potential role in influencing the risk of gastrointestinal cancers, marking a new direction in understanding DSC etiology.

Cathepsins are a widely expressed group of lysosomal proteases characterized by a broad spectrum of functions. Similar to other enzymes, they are vital for normal physiological functions such as innate immunity, apoptosis, angiogenesis, proliferation, and metastasis, underscoring their significance in cellular biology [5, 6]. Cathepsins have been known to remodel extracellular matrix (ECM) by specifically processing various proteins, including cytokines and chemokines, as well as shedding extracellular receptors and cell adhesion molecules [7–9]. Increased activity of cathepsins in the extracellular space is a recognized characteristic of various pathological conditions, including cancer and disorders affecting bone, joints, and the cardiovascular system [10, 11].

Cathepsins have long been closely linked with cancer research [12]. Pathologically alternated levels of cathepsins have been observed in a variety of cancers such as breast, lung, colon, pancreas, skin, prostate, bladder, ovary, and head and neck [8]. Nidhi Singh et al. reported heightened levels of cathepsin L in pancreatic cancer patients [13]. Similarly, increased levels of cathepsin L are found in hepatocellular carcinoma, usually correlated to worse prognostic outcomes [14]. Patients diagnosed with colorectal cancer exhibit elevated production and activity of cathepsin D, which may indicate a malignant condition of the large intestine [15]. At an experimental level, Zobeida Cruz-Monserrate et al. revealed the correlation of cathepsin E activity with pancreatic ductal adenocarcinoma tumors and precursor lesions, suggesting its potential as a molecular target and detection biomarker for pancreatic cancer [16]. These findings imply the intricate involvement of cathepsins in DSCs. Nevertheless, the roles of distinct cathepsins can differ significantly across various cancer types, and a holistic comprehension of the specific causalities between various cathepsin types and the etiologies of DSCs remains to be fully elucidated [17].

​In this investigation, we leveraged a subset of Mendelian randomization (MR) as a novel analytical framework to elucidate the potential causal linkages between cathepsins and digestive system malignancies. Specifically, employing a multivariable Mendelian randomization (MVMR) approach, we discerned divergent findings pertaining to hepatocellular carcinoma [18]. Mendelian Randomization (MR) is an emerging epidemiological method to infer causalities based on statistics. It leverages single nucleotide polymorphisms (SNPs) derived from genome-wide association studies (GWAS) as genetic instrumental variables (IVs) to elucidate causal relationships between specific exposures and outcomes [19]. Compared to traditional observational studies, an MR analysis is independent of errors caused by confounding or reverse causation, as genetic variations are fixed at conception and cannot be affected by exterior environment or personal lifestyles [20, 21]. In this study, we conducted univariable MR (UVMR), reverse MR, and multivariable MR (MVMR) analyses to examine the causal relationships between various cathepsin types and the risk of DSCs.

## Results

**Fig. 1:**
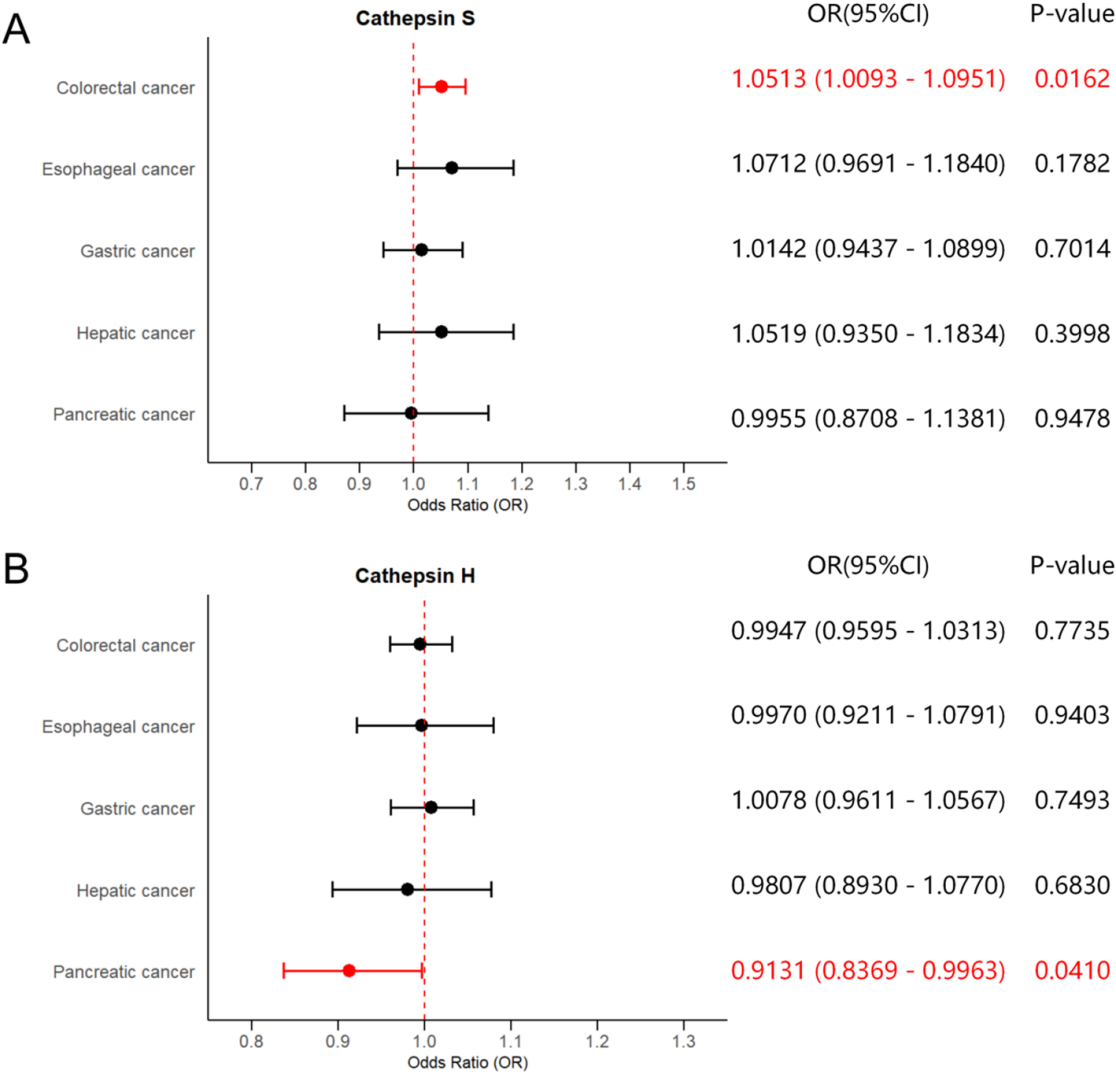
Forest plots of significant estimates of UVMR analyses. The IVW method was employed to explore the causalities of cathepsins on DSCs risk. **A** UVMR results of cathepsin S on DSCs risk; **B** UVMR results of cathepsin H on DSCs risk. DSC, digestive system cancer; IVW, inverse variance weighted; UVMR, univariable Mendelian randomization; OR, odds ratio; CI, confidence interval. Statistically significant results are indicated in red, with error bars representing 95% confidence intervals

Detailed results of UVMR are presented in Table 1. The estimates of the IVW method revealed that genetically predicted SNP-predicted cathepsin S expression were significantly positively associated with the risk of colorectal cancer (IVW: *p* = 0.0162, odds ratio (OR) = 1.0513, 95% confidence interval (CI) = 1.0093–1.0951). Although the weighted median method and MR-Egger model did not observe any significant causal effects, the directions of their estimates were consistent with that of the IVW method (weighted median: *p* = 0.0734, OR = 1.0606, 95%CI = 0.9944–1.1312; MR-Egger: *p* = 0.6693, OR = 1.0174, 95%CI = 0.9408–1.1003). Conversely, protective effects of cathepsin H on the risk of pancreatic cancer were observed (IVW: *p* = 0.0410, OR = 0.9131, 95% CI = 0.8369–0.9963). While the weighted median method and the MR-Egger model did not demonstrate any significant causal effects, the estimate directions aligned with those observed in the IVW method (weighted median: *p* = 0.1676, OR = 0.9322, 95%CI = 0.8437-1.0300; MR-Egger: *p* = 0.4116, OR = 0.9486, 95%CI = 0.8412–1.0697). Significant estimates of UVMR are presented in Fig. 1 as forest plots. Cochran’s Q, MR-Egger intercept, and MR-PRESSO global tests did not reveal any evidence of heterogeneity or horizontal pleiotropy. The results of the heterogeneity and pleiotropy tests for UVMR analyses are displayed in Supplementary Table 3. No causalities were found between the other types of cathepsins and any DSCs.

**Table 1:** Causal relationships of cathepsins on DSCs risk estimated by UVMR

Cathepsin	nSNP	IVW		Weighted median		MR-Egger	
		P-value	OR (95%CI)	P-value	OR (95%CI)	P-value	OR (95%CI)
*Cathepsin B*							
Colorectal cancer	20	0.3117	0.9712 (0.9176–1.0278)	0.7296	0.9863 (0.9121–1.0665)	0.8832	1.0098 (0.8888–1.1472)
Esophageal cancer	20	0.8524	1.0131 (0.8833–1.1619)	0.4287	0.9301 (0.7773–1.1129)	0.4920	0.8967 (0.6612–1.2161)
Gastric cancer	20	0.1630	0.9411 (0.8642–1.0249)	0.2899	0.9306 (0.8145–1.0632)	0.2144	0.8871 (0.7392–1.0646)
Hepatic cancer	20	0.6976	1.0306 (0.8852–1.1999)	0.3221	1.1114 (0.9017–1.3701)	0.7533	1.0544 (0.7615-1.4600)
Pancreatic cancer	20	0.7484	1.0208 (0.8999–1.1580)	0.9447	1.0066 (0.8356–1.2127)	0.4685	0.8971 (0.6731–1.1957)
*Cathepsin E*							
Colorectal cancer	11	0.5850	0.9825 (0.9224–1.0466)	0.7046	0.9841 (0.9060–1.0690)	0.9269	0.9929 (0.8554–1.1524)
Esophageal cancer	11	0.9577	0.9963 (0.8672–1.1446)	0.5603	0.9477 (0.7908–1.1357)	0.2828	0.8317 (0.6063–1.1410)
Gastric cancer	11	0.2277	0.9566 (0.8899–1.0281)	0.1306	0.9314 (0.8495–1.0213)	0.2852	0.9051 (0.7622–1.0749)
Hepatic cancer	11	0.2250	0.9173 (0.7979–1.0546)	0.3136	0.9054 (0.7462–1.0985)	0.2445	0.8106 (0.5824–1.1281)
Pancreatic cancer	11	0.6216	0.9607 (0.8191–1.1267)	0.7291	0.9655 (0.7913–1.1779)	0.5002	1.1221 (0.8136–1.5475)
*Cathepsin F*							
Colorectal cancer	11	0.1116	0.9553 (0.9030–1.0107)	0.5331	0.9788 (0.9152–1.0469)	0.6445	0.9625 (0.8228–1.1260)
Esophageal cancer	11	0.3896	0.9274 (0.7810–1.1012)	0.4378	0.9381 (0.7983–1.1024)	0.8256	1.0561 (0.6590–1.6924)
Gastric cancer	11	0.6651	1.0131 (0.9551–1.0747)	0.8494	1.0076 (0.9323–1.0890)	0.1189	1.1568 (0.9802–1.3653)
Hepatic cancer	11	0.6833	0.9681 (0.8285–1.1312)	0.5559	0.9561 (0.8234–1.1102)	0.1630	0.7261 (0.4805–1.0972)
Pancreatic cancer	11	0.0762	0.8871 (0.7771–1.0127)	0.0969	0.8504 (0.7024–1.0297)	0.2966	0.8253 (0.5876–1.1591)
*Cathepsin G*							
Colorectal cancer	12	0.4204	1.0343 (0.9528–1.1229)	0.1725	1.0725 (0.9699–1.1859)	0.5638	1.0679 (0.8607–1.3250)
Esophageal cancer	12	0.9341	0.9926 (0.8330–1.1829)	0.5483	1.0778 (0.8440–1.3763)	0.9051	0.9712 (0.6076–1.5523)
Gastric cancer	12	0.6023	0.9726 (0.8760–1.0798)	0.9393	1.0050 (0.8840–1.1425)	0.5840	1.0916 (0.8057–1.4791)
Hepatic cancer	12	0.1515	1.1466 (0.9511–1.3824)	0.4737	1.0857 (0.8670–1.3597)	0.5158	1.1985 (0.7076–2.0301)
Pancreatic cancer	12	0.4095	0.9284 (0.7781–1.1077)	0.2455	0.8680 (0.6836–1.1022)	0.3609	0.8173 (0.5407–1.2353)
*Cathepsin H*							
Colorectal cancer	11	0.7735	0.9947 (0.9595–1.0313)	0.9797	0.9995 (0.9647–1.0356)	0.8252	0.9940 (0.9437–1.0470)
Esophageal cancer	11	0.9403	0.9970 (0.9211–1.0791)	0.7611	1.0133 (0.9304–1.1036)	0.9740	0.9982 (0.8960–1.1120)
Gastric cancer	11	0.7493	1.0078 (0.9611–1.0567)	0.8819	1.0037 (0.9562–1.0535)	0.6611	0.9854 (0.9276–1.0501)
Hepatic cancer	11	0.6830	0.9807 (0.8930–1.0770)	0.7742	0.9872 (0.9045–1.0776)	0.8371	1.0140 (0.8913–1.1537)
Pancreatic cancer	11	0.0410	0.9131 (0.8369–0.9963)	0.1676	0.9322 (0.8437-1.0300)	0.4116	0.9486 (0.8412–1.0697)
*Cathepsin O*							
Colorectal cancer	12	0.9656	1.0016 (0.9323–1.0761)	0.7861	0.9876 (0.9023–1.0809)	0.7820	0.9694 (0.7823–1.2012)
Esophageal cancer	12	0.2556	1.1002 (0.9332–1.2971)	0.6428	1.0518 (0.8498–1.3018)	0.4397	1.2285 (0.7442–2.0281)
Gastric cancer	12	0.4197	0.9642 (0.8825–1.0535)	0.2912	0.9400 (0.8380–1.0545)	0.4755	1.1348 (0.8124–1.5852)
Hepatic cancer	12	0.6554	1.0391 (0.8779-1.2300)	0.2164	1.1486 (0.9221–1.4307)	0.9240	0.9686 (0.5112–1.8353)
Pancreatic cancer	12	0.9896	1.0012 (0.8398–1.1935)	0.7869	0.9679 (0.7642–1.2260)	0.4450	1.1984 (0.7672–1.8720)
*Cathepsin S*							
Colorectal cancer	24	0.0162	1.0513 (1.0093–1.0951)	0.0734	1.0606 (0.9944–1.1312)	0.6693	1.0174 (0.9408–1.1003)
Esophageal cancer	24	0.1782	1.0712 (0.9691–1.1840)	0.2657	1.0882 (0.9377–1.2630)	0.9603	1.0050 (0.8269–1.2215)
Gastric cancer	24	0.7014	1.0142 (0.9437–1.0899)	0.8217	1.0105 (0.9225–1.1069)	0.8118	1.0250 (0.8387–1.2526)
Hepatic cancer	24	0.3998	1.0519 (0.9350–1.1834)	0.0887	1.1487 (0.9792–1.3475)	0.9065	1.0189 (0.7485–1.3869)
Pancreatic cancer	24	0.9478	0.9955 (0.8708–1.1381)	0.9458	1.0052 (0.8663–1.1663)	0.8457	0.9759 (0.7653–1.2443)
*Cathepsin L2*							
Colorectal cancer	10	0.9273	0.9967 (0.9284-1.0700)	0.5267	1.0297 (0.9405–1.1274)	0.4869	0.9338 (0.7767–1.1227)
Esophageal cancer	10	0.5926	1.0483 (0.8819–1.2461)	0.5925	1.0667 (0.8421–1.3511)	0.4203	1.2191 (0.7719–1.9255)
Gastric cancer	9	0.2769	0.9452 (0.8540–1.0463)	0.7477	0.9785 (0.8574–1.1168)	0.2450	1.2431 (0.8883–1.7396)
Hepatic cancer	10	0.3296	0.9127 (0.7596–1.0967)	0.2956	0.8842 (0.7020–1.1136)	0.2951	1.3946 (0.7793–2.4958)
Pancreatic cancer	10	0.4776	1.0806 (0.8725–1.3382)	0.9506	1.0083 (0.7767–1.3088)	0.2485	1.3925 (0.8267–2.3454)
*Cathepsin Z*							
Colorectal cancer	13	0.1051	0.9561 (0.9055–1.0095)	0.0777	0.9480 (0.8933–1.0060)	0.2504	0.9407 (0.8521–1.0384)
Esophageal cancer	13	0.4652	0.9603 (0.8614–1.0705)	0.3461	0.9318 (0.8045–1.0793)	0.5531	0.9416 (0.7765–1.1418)
Gastric cancer	13	0.1998	1.0428 (0.9781–1.1117)	0.6184	1.0207 (0.9415–1.1066)	0.7078	0.9743 (0.8535–1.1123)
Hepatic cancer	13	0.4942	0.9581 (0.8476–1.0831)	0.0581	0.8637 (0.7423–1.0050)	0.0532	0.7712 (0.6096–0.9756)
Pancreatic cancer	13	0.4028	0.9301 (0.7848–1.1022)	0.4746	0.9328 (0.7708–1.1288)	0.3952	0.8774 (0.6567–1.1723)

Furthermore, reverse MR analyses were also conducted to assess the potential existence of reverse causality. Detailed results of reverse MR analyses are presented in Supplementary Table 4. No reverse causalities between any types of cathepsin and the risk of any DSCs were uncovered. The results of Cochran’s Q, MR-Egger, and MR-PRESSO global tests showed no signs of heterogeneity or horizontal pleiotropy. The results of the heterogeneity and pleiotropy tests for reverse MR analyses are displayed in Supplementary Table 5.

**Fig. 2:**
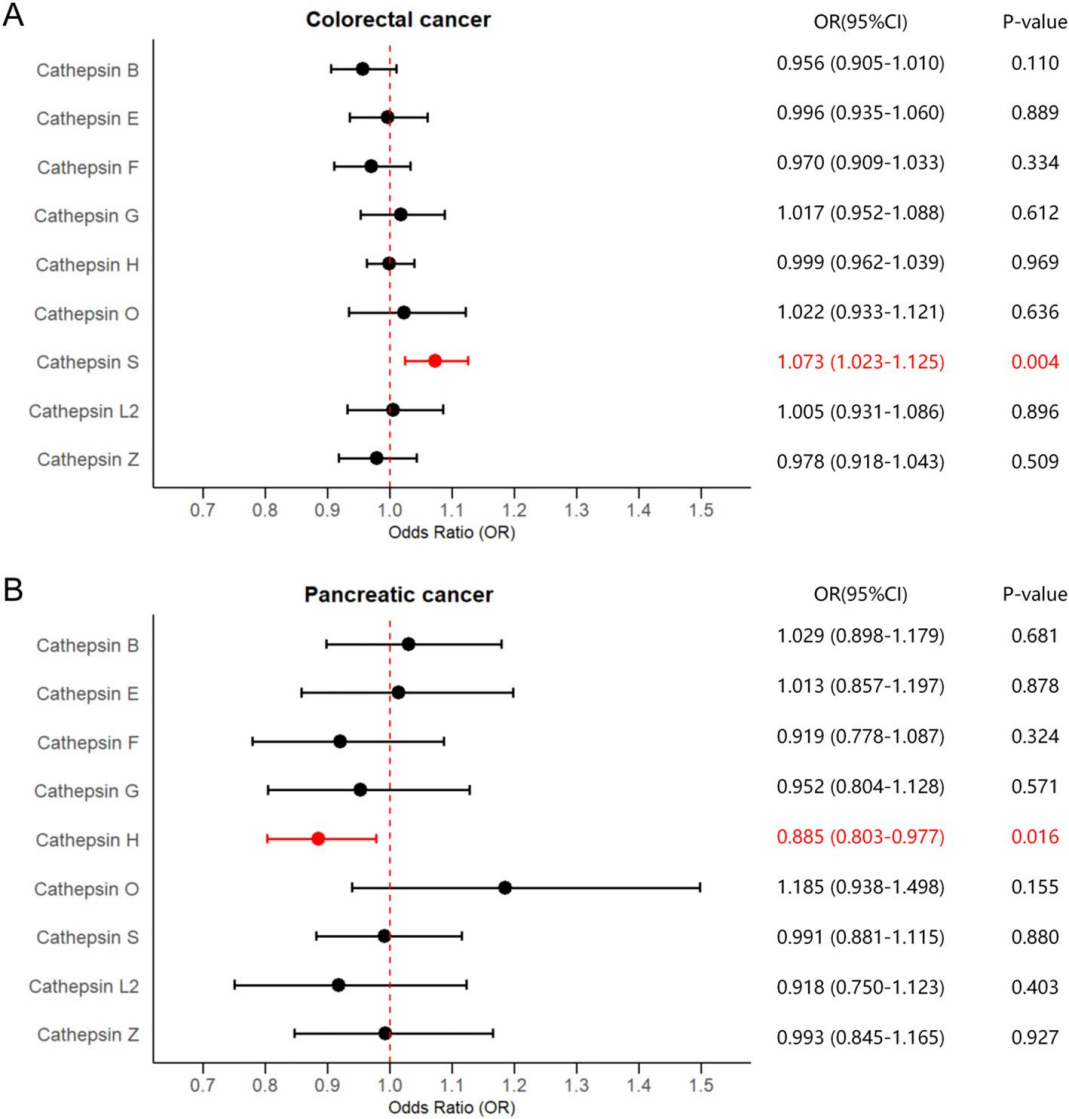
Forest plots of estimates of MVMR analyses. The IVW method was employed to explore the causalities of cathepsins on DSCs risk. **A** MVMR results of cathepsins on colorectal cancer risk; **B** MVMR results of cathepsins on pancreatic cancer risk. DSC, digestive system cancer; IVW, inverse variance weighted; MVMR, multivariable Mendelian randomization; OR, odds ratio; CI, confidence interval. Statistically significant results are indicated in red, with error bars representing 95% confidence intervals

Adjusting for the influence of the other cathepsins, MVMR analyses were also carried out to validate the results obtained from UVMR analyses. Detailed results of MVMR analyses and sensitivity analyses are shown in Supplementary Table 6. Consistent with UVMR, the results of MVMR indicated that higher SNP-predicted cathepsin S expression remained robustly associated with a raised risk of colorectal cancer after adjusting for the other types of cathepsins (IVW: *p* = 0.0040, OR = 1.0725, 95%CI = 1.0233–1.1252), which were further supported by the MR-Egger method (*p* = 0.0030, OR = 1.0736, 95%CI = 1.0243–1.1252). No heterogeneity or horizontal pleiotropy was detected in the estimate. The causal relationship between higher cathepsins H levels and decreased risk of pancreatic cancer (IVW: *p* = 0.0160, OR = 0.8851, 95%CI = 0.8025–0.9773) were steadily observed, which were corroborated by the MR-Egger method (*p* = 0.0110, OR = 0.8799, 95%CI = 0.7969–0.9714). No heterogeneity or horizontal pleiotropy was detected. Significant estimates of MVMR are presented in Fig. 2 as forest plots. No statistically significant causal relationships between the other types of cathepsins and DSCs were discovered in the MVMR analyses.

## Discussion

The progression of tumors is a highly intricate process, significantly influenced by proteolytic events, among which cathepsins have emerged as significant contributors, drawing substantial research interest [22]. Capitalizing on summary genetic statistics from two large-scale GWASs, this MR study aimed at deciphering the potential causal relationships between nine different types of cathepsins and the risk of diverse DSCs. To our knowledge, this study represented a pioneering effort in combining UVMR, reverse MR, and MVMR analyses to establish causalities between cathepsins and DSCs.Compared to previous studies investigating the causal relationships between cathepsins and digestive system cancers using Mendelian Randomization (MR), our study offers several methodological and analytical advancements. Firstly, we employed multivariable Mendelian randomization (MVMR) to account for potential pleiotropy and shared genetic architecture among cathepsins, which allows us to better isolate the independent effects of each cathepsin on cancer outcomes. This approach was not applied in earlier studies and enhances the robustness and interpretability of our findings.While previous research has included a broader set of cancer outcomes such as biliary tract cancer, our study focuses on a more rigorous methodological framework, which complements and builds upon the existing literature. The combination of MVMR, reverse MR, and sensitivity analyses strengthens the causal inference and offers novel insights into the potential role of cathepsins as biomarkers or therapeutic targets in digestive system cancers [18]. In summary, we discovered that cathepsin S was a risk factor for colorectal cancer. On the contrary, cathepsin H was indicated to have protective effects on the risk of pancreatic cancer. Our study found no evidence of reverse causality for both cathepsin S and cathepsin H.

The findings of this study indicated that elevated levels of cathepsin S were correlated to a higher risk of colorectal cancer. No evidence of heterogeneity, horizontal pleiotropy, or reverse causality was detected in this association. Cathepsins are a group of cysteine proteases known for their lysosomal activity and play vital roles in physiological functions such as protein turnover and processing [23]. They have been implicated in tumorigenesis, promoting a range of tumor-associated processes, including invasion, metastasis, and angiogenesis, and are widely considered significant contributors to the development and progression of various cancers [8, 12, 24]. With respect to cathepsin S, there is an increasing body of studies highlighting the connection between dysregulated expression of cathepsin S and disease aggressiveness in a spectrum of tumors, including prostate, gastric, and colorectal cancer [25–27]. Burden et al. first reported elevated cathepsin S expression in clinical samples obtained from colorectal cancer patients compared to those from normal colon tissues [26]. In an immunohistochemical analysis, it was identified that cathepsin S expression serves as a prognostic biomarker for colorectal cancer. Additionally, it has been identified as predictive of the response to adjuvant chemotherapy, suggesting the potential of cathepsin S to serve both as a prognostic indicator and as a novel biomarker for predicting the response to treatments in colorectal cancer patients [28].

Recent findings indicate that a plethora of tumor cells produce excessive cathepsin S, which has a significant impact on the tumor microenvironment (TME). This overproduction contributes to tumorigenesis and a metastatic phenotype via a range of mechanisms, including degrading ECM, promoting angiogenesis, and increasing inflammation [29–32]. In agreement with these mechanistic findings, the application of Fsn0503, a cathepsin S monoclonal antibody, was shown to reduce tumor invasion due to attenuated proteolytic activity of extracellular cathepsin S [26]. This finding is further supported in an in vivo study, where treatment of HCT116 xenograft colorectal tumors with Fsn0503 significantly hinders angiogenesis and tumor growth [26]. Cathepsin S is also recognized as a regulator of the transcription of CCL2, an important pro-inflammatory cytokine driving the recruitment of macrophages [33]. Experiments revealed that depletion of cathepsin S in tumors leads to reduction of CCL2 and tumor-associated macrophages, demonstrating the roles of cathepsin S in altering the cellular constitution of the TME and driving tumorigenesis [33].

Furthermore, our UVMR and MVMR analyses suggested that cathepsin H may have protective effects against pancreatic cancer. Though direct evidence regarding cathepsin H remains elusive, prior studies have delved into the latent mechanisms through which cathepsins interplay with DSCs, indicating that the roles of cathepsins in pancreatic cancer are possibly connected with their distinct influences on diverse pathophysiological processes, including ECM remodeling and tumor angiogenesis [34]. In addition, Cathepsin H may play a unique role in apoptosis, the process of programmed cell death critical for maintaining cellular homeostasis. Dysregulation of apoptosis is a hallmark in many cancers, including pancreatic cancer, leading to uncontrolled cell proliferation [34]. It has been shown that cathepsin H is involved in apoptosis by cleaving the pro-apoptotic protein Bid following selective lysosomal disruption [35]. This finding is further validated in an animal study reporting that the apoptotic index significantly increases when cathepsin H is genetically deleted in the homozygous knockout mouse models [36]. It is important to acknowledge that the number of specific studies on cathepsin H in pancreatic cancer is limited. The exact mechanisms by which cathepsin H influences the development and progression of pancreatic cancer remain to be discovered. Since the potential causalities of cathepsins on DSCs estimated in our MR analyses were restricted to a genetic level, future research, specifically focusing on the interplay of cathepsin H and DSCs, is necessary to provide more concrete evidence and understanding in this aspect.

This study represented a pioneering approach to investigating the relationships between various cathepsins and a spectrum of DSCs through MR analyses. It marked a significant stride towards unraveling genetic influences in gastrointestinal oncology. By integrating UVMR, reverse MR, and MVMR, this study provided a comprehensive perspective on the genetic interactions between cathepsins and DSCs. This multi-pronged strategy enhanced the validity of our findings through methodological diversity and cross-validation. Crucially, the reliance on genetic variants as IVs minimized confounding and mitigates the risk of reverse causation, yielding more reliable causal inferences. Most importantly, these insights bore substantial implications for the development of both diagnostic markers and therapeutic targets in DSCs, advancing the application of genetic knowledge in DSCs care.

Our study shed light on the relationship between cathepsins and DSCs, yet it’s important to recognize its limitations.

In this study, we adopted a genome-wide significance threshold of *p* < 5 × 10⁻⁶ for selecting instrumental variables, consistent with the original INTERVAL study, to accommodate the limited sample size of the proteomic GWAS. We acknowledge that applying a more stringent threshold (e.g., *p* < 5 × 10⁻⁸) could enhance instrument specificity. Although such an analysis was not performed in the current study, we plan to conduct a sensitivity analysis using this stricter threshold in future work to further assess the robustness of our findings.

Another important limitation of our study is the potential for weak instrument bias. The exposure GWAS used for cathepsin levels had a relatively modest sample size (*N* = 3,301), which may compromise the strength of the instrumental variables, particularly given the inclusion of nine distinct cathepsins in the analysis. While we selected SNPs reaching genome-wide significance, the F-statistics for these instruments were not initially reported, making it difficult to formally assess instrument strength—a critical factor for the validity of Mendelian Randomization (MR) analyses. To address this, we have now provided the F-statistics for each SNP in the revised results.

Additionally, the power to detect causal associations may be limited by the relatively small sample sizes of some outcome GWAS datasets, especially for hepatic cancer (*N* = 379) and esophageal cancer (*N* = 998). These constraints may have led to underpowered analyses, increasing the likelihood of false negatives. We have revised the Discussion to explicitly acknowledge these issues and emphasize the need for replication in larger and more diverse cohorts to validate our findings.

We acknowledge the value of further exploring the associations between individual SNPs or grouping SNPs based on their direction of effect (i.e., increasing or decreasing cathepsin expression) and cancer risk. Such analyses, possibly through functional studies or cohort-based investigations, could offer deeper insights into the biological roles of cathepsins. However, due to current limitations in available data and resources, we were unable to perform these additional analyses. This represents one of the limitations of our study. Moreover, The predominantly European ancestry of our study participants may limit the broader applicability of our findings across different ethnic groups. We fully acknowledge the importance of evaluating the generalizability of our results in diverse populations and hope to expand this research to include non-European ancestries in the future. Mendelian Randomization (MR) holds great promise for elucidating causal relationships in a global context. However, most current large-scale proteomic and GWAS datasets were initially developed and are most complete in populations of European descent. As a result, data for other ancestral groups remain limited and less comprehensive. Incorporating such data at this stage may introduce uncertainty and compromise the reliability of causal estimates. This represents a key limitation of our study and underscores the urgent need for more inclusive genetic research worldwide. Furthermore, our study serves as an initial exploration of the genetic associations between cathepsins and digestive system cancers (DSCs), highlighting the necessity of further investigations to bridge these genetic insights with clinical relevance across ethnically diverse populations.

Another limitation of our study is that we did not explicitly report the heritability of cathepsin expression levels. Although prior research [37]suggests that genetic variants account for a measurable proportion of variance in circulating protein levels, the heritability of cathepsins may still be modest. Given that MR analyses may be less robust when applied to exposures with low heritability, this remains a potential concern. Nevertheless, the genome-wide significant SNPs selected as instrumental variables demonstrated acceptable F-statistics, mitigating the risk of weak instrument bias to some extent.

Furthermore, although linkage disequilibrium score regression (LDSC) is a powerful approach for estimating genome-wide genetic correlation between traits, we were unable to perform LDSC due to the unavailability of full summary statistics for certain cathepsin subtypes. We acknowledge this as a limitation and suggest that future studies incorporate LDSC analysis to further validate the genetic relationships between cathepsins and digestive system cancers.

## Conclusion

In our study, we established a link between elevated SNP-predicted cathepsin S expression and an increased risk of colorectal cancer, while high cathepsin H levels might diminish pancreatic cancer risk. These findings paved the way for the development of both diagnostic markers and therapeutic targets, aiming to enhance the prediction and treatment of DSCs. Nevertheless, further research is imperative to effectively apply these findings in clinical practices and therapeutic approaches.

## Materials and methods

### Study design

**Fig. 3:**
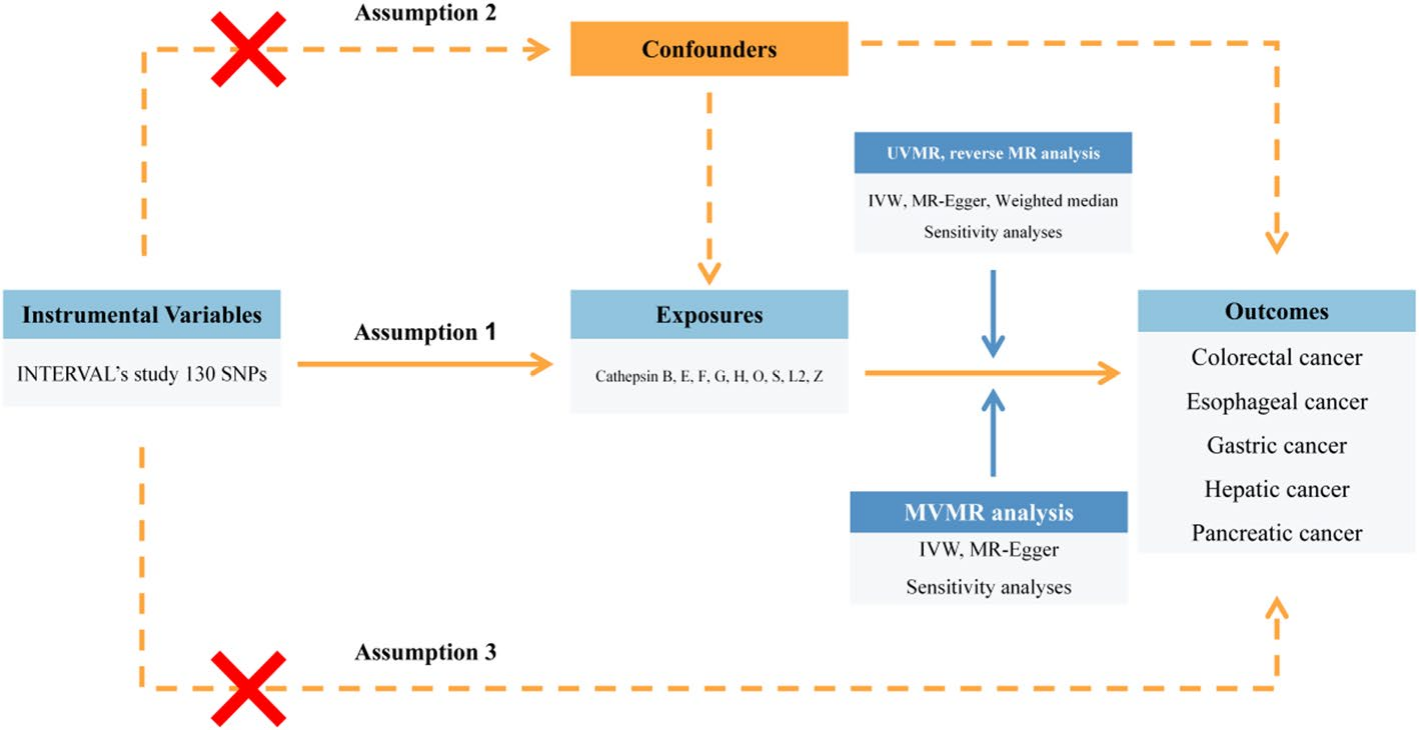
Flow chart of the entire study design. MR, Mendelian randomiazation; UVMR, univariable Mendelian randomization; MVMR, multivariable Mendelian randomization; SNP, single nucleotide polymorphism; IVW, inverse variance weighted

A detailed overview of the study design is shown in Fig. 3. Utilizing summary-level genetic data from two large-scale GWASs, we investigated the latent causalities of cathepsins on the risk of DSCs through UVMR, reverse MR, and MVMR analyses. A total of nine types of cathepsins (cathepsin B, E, F, G, H, O, S, L2, Z) were included as exposures, while five DSCs (esophageal cancer, gastric cancer, colorectal cancer, hepatic cancer, pancreatic cancer) were included as outcomes in this study. Three key assumptions were strictly followed to achieve reliable estimates in the MR analysis: (1) Genetic variants should exert a direct and statistically significant influence on exposure factors. (2) Genetic variants should solely impact the outcomes through their effects on the exposures. (3) Genetic variants should be strictly independent of any potential confounders [38, 39].

### Selection of instrumental variables for cathepsins

The genetic data on various cathepsins used in this study were sourced from the INTERVAL study, including a cohort of 3,301 European participants [40]. The study was approved by the National Research Ethics Service (11/EE/0538), with each participant’s informed consent provided. The data source of cathepsins is given in Supplementary Table 1. Primary criteria for selecting candidate SNPs as IVs in our study were established as follows: (1) SNPs should have P-values below the genome-wide significance threshold of 5e-6 as suggested in the original study, corresponding to the limitations of the sample size. (2) The SNPs should have a clump distance greater than 10,000 kb and an r2 value less than 0.001 to minimize the risk of potential linkage disequilibrium (LD). In addition, we cross-referenced each selected SNP in the PhenoScanner database to confirm and exclude any SNP susceptible to any known confounders [41]. Data of SNPs selected as IVs are detailed in Supplementary Table 2.

### Selection of instrumental variables for DSCs

IVs associated with DSCs were also retrieved to conduct reverse MR analyses. Summary statistics of esophageal cancer (998 cases, 475,308 controls), gastric cancer (1,029 cases, 475,087 controls), colorectal cancer (6,581 cases, 463,421 controls), hepatic cancer (379 cases, 475,259 controls), and pancreatic cancer (1,196 cases, 475,049 controls) were extracted from a GWAS published by Sakaue et al., with the major population of European descent [42]. Similarly, IVs for DSCs were reached through the steps mentioned above. Data source of DSCs is displayed in supplementary Table 1. All participants granted informed written consent, and all research endeavors underwent thorough review and approval by the ethics review committees at the involved institutions. Phenotypes used in this study were available online at the Integrative Epidemiology Unit (IEU) OpenGWAS Project website (https://gwas.mrcieu.ac.uk).

### Statistical analyses

Multiple methods were utilized to conduct UVMR analyses, encompassing inverse variance weighted (IVW), weighted median, and MR-Egger. Each method operates on different underlying assumptions and addresses the pleiotropic effects through various strategies [43–45]. IVW is a primary technique frequently employed in MR studies. It aggregates Wald ratios from each SNP to produce a combined estimate, which grants it considerable statistical power [45, 46]. Therefore, IVW was considered the main analysis to investigate the causal effects in this study. The weighted median and MR-Egger methods were also adopted as auxiliary methods to examine the robustness of our findings. The weighted median method involves calculating a median, where each individual MR estimate is weighted based on its precision. It allows the inclusion of certain invalid variants as IVs, provided that at least half of the IVs are valid [44]. Meanwhile, the MR-Egger is a method that performs weighted linear regression of the associations between SNP outcomes and SNP exposures. It provides an unbiased causal estimate even when all IVs are invalid [43]. The MR-Egger technique can detect and correct horizontal pleiotropy despite its relatively low accuracy of estimation [47]. When no horizontal pleiotropy was present, the main causal effects in this MR study were determined using results from the IVW method with random effects. Conversely, in cases where horizontal pleiotropy was identified, the MR-Egger method’s results were utilized to assess the main causal effects. Despite the relatively low statistical efficiency of the weighted median and the MR-Egger methods, these approaches were instrumental in providing crucial insights and contributed significantly to a comprehensive evaluation of the consistency and reliability of the results obtained in our research [48]. Therefore, we mainly focused on whether the magnitude and direction of effect estimates were consistent across methods. Statistically, a p-value below 0.05 was considered of statistical significance. Simultaneously, we performed sensitivity analyses to evaluate the potential impact of heterogeneity and pleiotropic effects on our estimates, aiming to ensure the credibility and robustness of the results. The Cochran’s Q test was conducted to evaluate possible heterogeneity in the estimates, with a p-value less than 0.05 indicating significant heterogeneity [49]. To detect horizontal pleiotropy, we conducted the MR-Egger intercept test, with a p-value less than 0.05 for the MR-Egger intercept suggesting the presence of pleiotropy [47]. The MR Pleiotropy RESidual Sum and Outlier (MR-PRESSO) test was conducted to test horizontal pleiotropy by identifying and removing any outlier IVs [50]. The Leave-one-out (LOO) test was applied to identify any SNPs with extreme influence on the estimates. UVMR analyses were conducted using the “TwoSampleMR” R package [51].

MVMR analyses were also implemented to provide a comprehensive assessment of the estimated causal relationships [52]. In MVMR analyses, IVs associated with multiple cathepsins were collectively considered to estimate the direct causal effect of each exposure on a single DSC [53]. MVMR analyses were conducted with the “MendelianRandomization” R package [54]. Additionally, we employed reverse MR analyses to mitigate potential biases from the UVMR analyses, treating DSCs as exposures and cathepsins as outcomes, which was instrumental in identifying possible reverse causality. In reverse MR analyses, summary statistics of DSCs and cathepsins were obtained from the same GWAS datasets as mentioned above. Reverse MR analyses were conducted with the “TwoSampleMR” R package. All statistical analyses were performed using R software version 4.3.1.

## Supplementary Information

Supplementary Material 1

## Data Availability

The data used in this study are available in public databases the Integrative Epidemiology Unit (IEU) OpenGWAS Project website (https://gwas.mrcieu.ac.uk) and the GWAS Catalog website (https://www.ebi.ac.uk/gwas). Detailed dataset descriptions, summary data of key results, and the information underpinning Fig. 1 and Fig. 2 are available in Supplementary Tables 1-8.
